# Four-day antithrombin therapy does not seem to attenuate hypercoagulability in patients suffering from sepsis

**DOI:** 10.1186/cc5098

**Published:** 2006-11-15

**Authors:** Christopher Gonano, Christian Sitzwohl, Eva Meitner, Christian Weinstabl, Stephan C Kettner

**Affiliations:** 1 Department of Anesthesiology and General Intensive Care, Medical University of Vienna, Waehringer Gürtel 18-20, A-1090 Vienna, Austria; 2 Austrian Anesthesiology and Critical Care Foundation, Vienna, Austria

## Abstract

**Introduction:**

Sepsis activates the coagulation system and frequently causes hypercoagulability, which is not detected by routine coagulation tests. A reliable method to evaluate hypercoagulability is thromboelastography (TEG), but this has not so far been used to investigate sepsis-induced hypercoagulability. Antithrombin (AT) in plasma of septic patients is decreased, and administration of AT may therefore reduce the acquired hypercoagulability. Not clear, however, is to what extent supraphysiologic plasma levels of AT decrease the acute hypercoagulability in septic patients. The present study investigates the coagulation profile of septic patients before and during four day high-dose AT therapy.

**Methods:**

Patients with severe sepsis were randomly assigned to receive either 6,000 IU AT as a bolus infusion followed by a maintenance dose of 250 IU/hour over four days (*n *= 17) or placebo (*n *= 16). TEG, platelet count, plasma fibrinogen levels, prothrombin time and activated partial thromboplastin time were assessed at baseline and daily during AT therapy.

**Results:**

TEG showed a hypercoagulability in both groups at baseline, which was neither reversed by bolus or by maintenance doses of AT. The hypercoagulability was mainly caused by increased plasma fibrinogen, and to a lesser extent by platelets. Plasmatic coagulation as assessed by the prothrombin time and activated partial thromboplastin time was similar in both groups, and did not change during the study period.

**Conclusion:**

The current study shows a distinct hypercoagulability in patients suffering from severe sepsis, which was not reversed by high-dose AT treatment over four days. This finding supports recent data showing that modulation of coagulatory activation in septic patients by AT does not occur before one week of therapy.

**Trial registration**: Current Control Trials ISRCTN22931023

## Introduction

Sepsis activates the host's defense system by initiating the release of a complex network of proinflammatory and anti-inflammatory cytokines. The septic process frequently induces intravascular coagulation, which activates endogenous anticoagulants and the fibrinolytic system. As a consequence coagulation inhibitors are consumed, and fibrinolysis is inhibited by the production of plasminogen activator inhibitor 1 [1]. Hypercoagulability develops in septic patients, resulting in enhanced thrombin generation, thrombin activation, and fibrin formation. Routine coagulation tests, such as the prothrombin time and the activated partial thromboplastin time, do not reflect this state, as they are sensitive for coagulation defects not for hypercoagulability [2]. A reliable method to evaluate hypercoagulability is thromboelastography (TEG) [3-6]. No study, however, has so far investigated hypercoagulability in patients suffering from sepsis with the use of TEG.

Physiologically, three main inhibitors are involved in the host defense against the activation of coagulation: the tissue factor, the protein C system, and antithrombin (AT). The concentration of AT in plasma of septic patients is decreased, and this is a predictor of an unfavorable prognosis [7]. Open-labeled and phase II trials showed that administration of AT concentrates may improve the outcome of sepsis due to the reduction of hypercoagulability and due to the anti-inflammatory properties of AT [8-10]. One large phase III study, however, failed to improve the outcome of septic patients, perhaps due to study biases or drug interaction [11,12].

In studies using high-dose AT therapy in septic patients, either the effects of AT on coagulation have not been described in detail [8,9,11] or coagulation markers, such as protein C and prothrombin activity, have been investigated [10,13]. To what extent supraphysiologic levels of AT decrease the acute hypercoagulability in septic patients is therefore unknown.

We hypothesized that TEG can assess which patients suffering from sepsis show hypercoagulability, and that high-dose AT therapy may reduce this hypercoagulability. Accordingly, we investigated TEG, plasmatic coagulation tests, plasma levels of fibrinogen, and platelet counts in septic patients before and during high-dose AT therapy.

## Methods

This study was performed as an addition to a double-blind, placebo-controlled, multicenter clinical phase III trial in patients with severe sepsis (the KyberSept trial) [11]. In accordance with the Institutional Review Board of the University of Vienna, patients were included in the study and their written informed consent was obtained after adequate recovery.

Patients suffering from severe sepsis were assigned by a telephone randomization service to receive either AT (Aventis Behring GmbH, Marburg, Germany) (*n *= 17) or placebo solution (1% human albumin) (*n *= 16). The treatment group received 6,000 IU AT as a bolus infusion followed by a maintenance dose of 250 IU/hour over four days. Standard therapy, such as antimicrobial therapy, respiratory or hemodynamic support, and fluid administration, was not influenced by the study and was at the physicians' discretion.

Patients with suspected severe sepsis were enrolled if they fulfilled the following criteria within a six hour time window prior to randomization: clinical evidence of sepsis with a suspected source of infection, body temperature (rectally or core) >38.5°C or <35.5°C, and leukocyte count >10,000/μl or <3,500/μl. Additionally, three out of the following six criteria had to be met: heart rate >100 beats/min, tachypnea >24 breaths/min or mechanical ventilation because of septic indication, systolic blood pressure <90 mmHg despite sufficient fluid replacement or the need for vasoactive agents to maintain systolic blood pressure >90 mmHg, thrombocytopenia with platelet counts <100,000/ml, elevated lactate levels or metabolic acidosis (that is to say, pH <7.3 or base excess above -10 mmol/l) not secondary to respiratory alkalosis, and oliguria with urine output <20 ml/hour despite sufficient fluid replacement. The exclusion criteria were, among others [11], pregnancy and/or breastfeeding, presence of a condition other than sepsis anticipated to be fatal within 28 days, history of hypersensitivity to the study medication, treatment with an AT concentrate within the previous 48 hours, treatment with heparin exceeding 10,000 IU/day low-molecular-weight heparin, known bleeding disorders or ongoing massive surgical bleedings, nonsteroidal anti-inflammatory drugs in anti-inflammatory doses within the previous two days, platelet count <30,000 ml, pre-existing dialysis-dependent renal failure, end-stage liver disease, or enrollment in another clinical trial within the previous 30 days.

Routine laboratory measurements were carried out as usual. The coagulation profile was assessed at least three times a day during treatment with the study medication. Routine AT measurements during the first 14 days of the study were not performed, to ensure the double-blinded fashion of the study. Samples for determination of AT (activity and antigen) were drawn before the bolus infusion, 24 hours after the start of bolus infusion, and then daily. These samples were centrifuged at 4°C and stored at -80°C for central measurements. The AT activity was provided by Aventis Behring GmbH after trial finalization. The platelet count, plasma fibrinogen levels, prothrombin time, and activated partial thromboplastin time were assessed at baseline and three times daily.

In addition to standard coagulation tests we performed two thromboelastograph scans before the bolus infusion and then daily (TEG^®^; Haemoscope, Skokie, IL, USA), each using 300 μl whole blood recalcified with 40 μl of 0.645% CaCl_2_. TEG measurements were performed after incubating the blood samples with heparinase, to exclude heparin effects on TEG. The antibody fragment abciximab (ReoPro^®^; Centocor, Leiden, The Netherlands) was added to one assay to evaluate platelet function [14,15]. TEG permits a reliable, global assessment of hemostatic function correlating to routine coagulation tests and, most importantly, to postoperative blood loss and the incidence of thrombotic complications [5,16]. Liquid whole blood transmits little or no torque in TEG, producing no amplitude on the TEG tracing even in blood samples with high viscosity [17]. As the blood clots, fibers composed of fibrin and platelets form, producing an increasing amplitude. Platelets provide a phospholipid surface for coagulation reactions in the standard TEG tracing and thus promote the formation of fibrin [17]. Furthermore, platelets bind to fibrinogen and modulate the viscoelastic properties of the clot via the platelet surface receptor glycoprotein IIb/IIIa [15]. The glycoprotein IIb/IIIa receptor can be sufficiently inhibited by the antibody-fragment abciximab. Fibrinogen is soluble until thrombin binds to the central region, which produces proteolysis at the N-terminus, releasing fibrinopeptide A and B and fibrin monomer. This release process exposes other regions of the molecule to interact with other activated fibrin molecules for polymerization of the fibrin network. The end point of this cascade is fibrin.

The reaction time (*r*), coagulation time (*k*), alpha angle (α), maximum amplitude (MA), and abciximab MA were assessed. Values for *r *and *k *are expressed in millimeters – as the chart speed is 2 mm/min, the time in minutes is equal to the distance in millimeters divided by two (normal ranges: *r *= 10–19 mm, *k *= 4–10 mm). The α value measures the speed of fibrin build-up and cross-linking, which resembles speed of clot strengthening (normal range: α = 44–56°). The MA measures the maximal clot strength, which is dependent on platelet function and, to a lesser extent, on fibrinogen level (normal range: MA = 50–64 mm). Whereas the correlation between standard MA and fibrinogen levels is usually weak, the modification of TEG with the antibody fragment abciximab results in a good correlation between fibrinogen levels and abciximab-modified MA [15]. Because the resulting MA of the abciximab-modified TEG is an estimation of the contribution of fibrinogen to clot strength, the difference of the standard MA and the abciximab-modified MA primarily reflects platelet function [15]. The TEG tracing of hypercoagulable blood typically shows a shorter reaction time, with a higher MA and a steeper α value than normal.

### Statistical analysis

An unpaired Mann–Whitney nonparametric test with Bonferroni correction for multiple testing was performed to compare for differences between the AT and placebo group. *P *< 0.05 was considered statistically significant, and all data are presented as the mean ± standard deviation or as the median (minimum–maximum) unless otherwise indicated.

## Results

Demographic data were similar among the groups (Table 1). None of the patients was on corticosteroid replacement therapy, as this was not a standard therapy for sepsis in our institution at that time. Two patients in each group received continuous hemofiltration during the AT treatment. All patients received noradrenalin, despite adequate volume resuscitation. Coagulation profiles as assessed by TEG showed a hypercoagulability in both groups of the included septic patients at baseline (Table 2). This hypercoagulability comprised all measured TEG parameters, since *r *and *k *were decreased and α and MA were increased compared with normal values. Both plasmatic and cellular hemostasis therefore showed hyperreactivity. High doses of AT did not reverse this hypercoagulability, and caused only a slight increase in the reaction time (Table 2). During the four days of treatment with AT the hypercoagulability assessed by TEG remained similar, although plasma levels of AT reached supranormal values.

We assessed the contribution of platelets and fibrinogen to hypercoagulability using the abciximab-modified MA. Hypercoagulability was mainly caused by the activity of plasma fibrinogen. The platelets also showed hyperreactivity, but contributed to hypercoagulability to a lesser extent than did plasma fibrinogen.

Plasmatic coagulation as assessed by the prothrombin time and the activated partial thromboplastin time was similar in both groups, and did not change during the study period. In contrast to the TEG parameters, the plasmatic coagulation tests were at the lower range of normal values or were even prolonged.

## Discussion

We investigated the effects of a four day treatment with high doses of AT on coagulation in septic patients. Our study shows a distinct hypercoagulability in patients suffering from sepsis as assessed by TEG. This hypercoagulability was not influenced by high doses of AT administered over four days, and treatment with AT influenced neither TEG parameters nor standard coagulation tests.

The high sensitivity of TEG to hypercoagulability has been described in numerous studies [3-5,15], and it is not surprising that we found a distinct hypercoagulability in patients suffering from severe sepsis. TEG has not so far been used to investigate sepsis-related hypercoagulability, but coagulation disturbances leading to hypercoagulability during sepsis are well described [1].

Surprising is the finding that high doses of AT hardly influence hypercoagulability, as assessed by TEG, in septic patients. The sensitivity of TEG to plasma levels of AT has been shown recently [18,19]. Hypercoagulability is attenuated in TEG when exogenous AT is administered to reach normal ranges of AT. It is remarkable, therefore, that the high-dose treatment with AT over four days, which caused supranormal plasma levels of AT, hardly affected the TEG variables.

High-dose AT therapy in septic patients has been investigated in several studies. Although phase II trials showed that administration of AT concentrates may improve the outcome of sepsis [8-10], the treatment with AT for four days did not reduce the overall 28-day mortality rates compared with placebo in a large phase III study [11]. Besides other factors [12], an insufficient dosage and duration of AT therapy could have been responsible for the negative results. In a recent study, prolonged duration of AT therapy guided by the actual activity, instead of a predefined dose, resulted in an effective modulation of coagulatory activation [13]. The effects of AT were thereby not evident until one week of therapy. Additionally, the selection of septic patients who may profit from AT therapy seems to be important. Patients with AT activity below 70% or patients undergoing continuous renal replacement therapies may profit most [20]. Our data seem to confirm these findings, as we did not find alterations of coagulation parameters during the four day treatment with high doses of AT in a population of septic patients not selected by AT activity or a need for renal replacement therapy.

The patients in the AT group were well matched to the control group concerning demographic data, coagulation parameters, and the extent of organ failure. The main finding of this study results from comparison of coagulation profiles within the AT group, which showed mild attenuation of hypercoagulability after bolus application of AT and no differences in the later time course. The control group was investigated to show the time course of hypercoagulability in septic patients without AT therapy. The finding that coagulation profiles do not differ between the AT group and the placebo group from the second day of treatment may imply that the selected dose of AT was not sufficient to reduce hypercoagulability and/or that a four day treatment is insufficient.

The abciximab-modified MA showed that hypercoagulability was mainly caused by the activity of plasma fibrinogen, and to a lesser extent by platelet hyperreactivity. Owing to the complex inclusion/exclusion criteria of the KyberSept trial, we may have excluded the patients who might have profited most from AT therapy. A recent retrospective analysis of the KyberSept trial actually showed that high-dose AT without concomitant heparin in septic patients with overt and nonovert disseminated intravascular coagulation resulted in a mortality reduction [21]. Many clinicians in our center administered AT when the AT level was below 40%, and, in addition, the indications for continuous renal replacement therapies with heparin anticoagulation are liberal. Both interventions led to the exclusion of numerous patients, most of whom had severe coagulation abnormalities. The platelet count was actually normal in most patients at inclusion. We therefore assume that we excluded patients with the strongest platelet activation, whereas the fibrinogen levels were generally increased at inclusion (Table 1).

Low-dose low-molecular-weight heparin was given subcutaneously in the present study to prevent thromboembolic complications, with similar dosing in both groups. As TEG is highly sensitive to all types of heparin, either unfractionated heparin or low-molecular-weight heparin [22], the TEG measurements were performed after incubating the blood samples with heparinase, to exclude heparin effects on TEG. The effects of heparinase *per se *are minor and, as all measurements were performed in an identical manner, incubation with heparinase should not have altered our results.

The limitation of the present study is the small number of included patients. The main finding of the study, however, was that AT therapy had hardly any effect on coagulation as assessed by TEG. Although one could speculate that the differences in TEG variables may become statistically significant with more patients included, the clinical significance seems questionable as TEG measurements both before and after administration of the AT bolus show a distinct hypercoagulability.

## Conclusion

The current study shows a distinct hypercoagulability in patients suffering from severe sepsis, which is not attenuated by administration of high doses of AT over four days. This finding supports recent data showing that prolonged duration of AT therapy guided by the actual activity, instead of a predefined dose, can result in the modulation of coagulatory activation after one week of therapy.

## Key messages

• Patients suffering from severe sepsis show a distinct hypercoagulability, which can be assessed by TEG.

• High-dose antithrombin therapy for four days does not attenuate this hypercoagulability.

## Abbreviations

α = alpha angle; AT = antithrombin; *k *= coagulation time; MA = maximum amplitude; *r *= reaction time; TEG = thromboelastography.

## Competing interests

The authors declare that they have no competing interests.

## Authors' contributions

CG and SCK conceived of the study and drafted the manuscript. CS performed the statistical analysis and helped to draft the manuscript. EM ran the protocol, helped in the statistical analysis, and drafted the manuscript. CW participated in design and helped to draft the manuscript. All authors read and approved the final version of this manuscript.

## Figures and Tables

**Table 1:** Demographic data of patients suffering from sepsis receiving either placebo or high-dose antithrombin therapy

	Placebo	Antithrombin
Number of patients (*n*)	16	17
Age (years)	56 ± 17	51 ± 19
Height (cm)	175 ± 4	169 ± 9
Weight (kg)	80.7 ± 22.5	75.9 ± 22.4
4-day survival rate (*n*)	16	16
28-day survival rate (*n*)	11	12
Acute Pathophysiology and Chronic Health Evaluation II score	52 ± 9.5	53 ± 12.6
Low-molecular-weight heparin at inclusion (IU/day)	6042 ± 1982	6388 ± 2205

Data presented as the mean ± standard deviation or *n*.

**Table 2:** Coagulation parameters during the study

	At baseline		After bolus application		At end of treatment period	
	
	Placebo	Antithrombin	Placebo	Antithrombin	Placebo	Antithrombin
Prothrombin time (%)	70 (38–95)	60 (38–103)	60 (39–84)	60 (38–82)	77 (5–10)	64 (5–9)
Activated partial thromboplastin time (s)	42 (27–53)	46 (33–60)	44 (32–67)	53 (34–77)	44 (29–74)	47 (34–95)
Fibrinogen (mg/dl)	587 (262–821)	557 (229–841)	595 (283–770)	505 (313–704)	622 (115–842)	538 (200–868)
Platelet count (1,000/ml)	225 (86–581)	179 (71–742)	170 (81–358)	154 (61–730)	199 (63–418)	115 (14–475)
Antithrombin (%)	68 (31–113)	55 (27–99)	54 (22–108)	110* (43–350)	86 (29–87)	141* (110–149)
Reaction time (mm)	7 (6–15)	9 (6–16)	8 (5–15)	11 (7–25)	8 (5–19)	11 (8–18)
Coagulation time (mm)	3 (2–4)	3 (2–8)	3 (2–4)	3 (2–7)	2 (1–8)	4 (3–10)
Alpha angle (°)	74 (64–80)	71 (50–80)	69 (64–81)	70 (36–79)	76 (43–81)	64 (46–76)
Maximum amplitude (mm)	69 (57–79)	66 (49–81)	66 (60–74)	70 (49–83)	71 (38–82)	72 (38–82)
Abciximab maximum amplitude (mm)	29 (15–49)	29 (13–49)	29 (23–45)	28 (12–45)	33 (14–48)	31 (7–48)

Data presented as the median (minimum–maximum). Normal ranges: prothrombin time = 75–140%, activated partial thromboplastin time = 27–41 s, reaction time = 10–19 mm, coagulation time = 4–10 mm, alpha angle = 44–56°, maximum amplitude = 50–64 mm. *P ≤ 0.05 compared with placebo.
